# Lack of association between vaccine‐induced immune thrombocytopenia and thrombosis and HLA loci in a large cohort

**DOI:** 10.1111/bjh.70419

**Published:** 2026-03-09

**Authors:** Linda Schönborn, Ana Tzvetkova, Thomas Thiele, Uwe Völker, Sabine Ameling, Sören Franzenburg, Lars Kaderali, Andreas Greinacher

**Affiliations:** ^1^ Department of Transfusion Medicine Universitätsmedizin Greifswald Greifswald Germany; ^2^ Department of Functional Genomics Universitätsmedizin Greifswald Greifswald Germany; ^3^ Institute of Clinical Molecular Biology Christian‐Albrechts‐University of Kiel Kiel Germany; ^4^ Institute of Bioinformatics Universitätsmedizin Greifswald Greifswald Germany

**Keywords:** adenovirus, adverse effects, HLA, immunogenetics, platelet factor 4, thrombocytopenia, thrombosis, vaccine, VITT

To the Editor,

Vaccine‐induced immune thrombocytopenia and thrombosis (VITT) is a rare but severe adverse event following adenoviral vector‐based vaccination against COVID‐19^1^, ^2^, ^3^ and after natural adenovirus infection.^4^, ^5^, ^6^ VITT is caused by platelet‐activating antibodies against platelet factor 4 (PF4) and subsequent platelet activation. Recent publications by Petito et al.^7^ and commentary by Arnold et al.^8^ have suggested a potential role for Human Leukocyte Antigen (HLA) polymorphisms in predisposing individuals to VITT. Three HLA class II alleles were detected with significantly higher frequency in 16 VITT patients compared with 198 Italian controls: HLA‐DPB1*17:01 (0.125 in VITT vs. 0.002 in controls, *p* = 0.0009), HLA‐DQA1*05:01 (0.375 in VITT vs. 0.085 in controls, *p* = 0.00015) and HLA‐DRB1*11:04 (0.218 in VITT vs. 0.053 in controls, *p* = 0.03380). We aimed to test whether previously reported HLA class II alleles, or any common HLA alleles, are associated with susceptibility to VITT in an independent, larger cohort.

The study included 100 Caucasian patients diagnosed with VITT according to established criteria,^9^, ^10^ described in detail by Schönborn et al.^11^, ^12^ Healthy Caucasians (*n* = 193) recruited from the population‐wide study of health in Pomerania (SHIP)^13^ in Northern Germany served as control. All participants (VITT patients and healthy subjects) provided informed consent, and the study was approved by the Ethics committee of the Universitätsmedizin Greifswald.

DNA of 100 patients with confirmed VITT was manually extracted from 100 to 200 μL buffy coat (including PBMCs) using a HMW MagAttract Kit (Qiagen, Hilden, Germany). High molecular weight DNA was re‐suspended in 80–100 μL elution buffer AE (10 mM Tris‐Cl, 0.5 mM EDTA pH 9.0). Input genomic DNA was quantified using a Quantifluor dsDNA kit (Promega, Fitchberg, USA) and ranged from 19 to 83 ng/μL. Quality of genomic DNA was determined using a Genomic DNA Screen Tape on an Agilent Tape Station 4200 (Agilent, Santa Clara, USA). DNA Integrity Numbers (DIN) ranged from 6.8 to 9.8. As input for the TruSeq DNA PCR‐free kit (Illumina, San Diego, USA), 1‐μg DNA was used according to the manufacturers reference guide #1000000039279 v00. Resulting libraries were inspected on the TapeStation 4200 with the D5000 Screen Tape (Agilent, Santa Clara, USA) and quantified using quantitative polymerase chain reaction (qPCR) with a KAPA Library Quantification Kit (Roche, Basel, Switzerland). Libraries were sequenced on an Illumina NovaSeq 6000 S4 Flowcell using 150 bp paired‐end reads with a final loading concentration of 300 pM and 22 samples per Flowcell.

To reconstruct the HLA HLA‐A, ‐B, ‐C, ‐DRB1, ‐DQA1, ‐DQB1 and ‐DPB1 loci, we applied the HLA*LA software^14^ with default parameters. Frequencies of HLA alleles were compared between VITT patients and controls using Fisher's exact test. Adjustments for multiple testing were performed using the Benjamini–Hochberg procedure.

Table 1 presents the frequencies of common HLA alleles in VITT patients and controls. No statistically significant differences were observed between the two groups after correction for multiple comparisons with the exception of HLA A*31:01:02G, which was present in 12 of 100 VITT patients but only in four of 193 controls (*p* = 0.027).

**TABLE 1:** Percentages of HLA‐A, ‐B, ‐C, ‐DP, ‐DQ and ‐DR allele frequencies in 100 VITT patients compared to 193 healthy controls.

	Allele	VITT, *n* = 100 (%)	Control, *n* = 193 (%)	FDR
HLA‐A	A*01:01:01G	12.5	12.2	1
	A*01:02	0	0.3	1
	A*02:01:01G	27	32.1	1
	A*02:03:01G	0.5	0	1
	A*02:05:01G	1	0.5	1
	A*02:06:01G	0.5	0.3	1
	A*02:245	0	0.3	1
	A*02:35:01	0	0.3	1
	A*03:01:01G	16	14.5	1
	A*03:36N	0.5	0	1
	A*11:01:01G	4.5	5.2	1
	A*11:190	0	0.3	1
	A*23:01:01G	2	2.6	1
	A*24:02:01G	10	8.3	1
	A*24:03:01G	0	0.3	1
	A*24:07:01	0.5	0	1
	A*25:01:01G	1	3.4	1
	A*26:01:01G	3	4.9	1
	A*29:01:01G	0	0.3	1
	A*29:02:01G	3.5	3.1	1
	A*30:01:01G	1	0.8	1
	A*30:02:01G	0.5	0.5	1
	A*31:01:02G	6	1	0.027
	A*32:01:01G	2.5	2.3	1
	A*33:01:01	1	0.8	1
	A*33:03:01G	0	0.3	1
	A*33:27	0	0.3	1
	A*66:01:01G	0	0.8	1
	A*68:01:01G	2	1.6	1
	A*68:01:02G	3.5	2.3	1
	A*68:02:01G	1	0.8	1
HLA‐B	B*07:02:01G	9	11.7	1
	B*07:05:01;B*07:06	0.5	0.5	1
	B*07:118	0	0.3	1
	B*08:01:01G	8.5	9.6	1
	B*13:01:01G	0.5	0	1
	B*13:02:01G	4.5	1.6	1
	B*14:02:01	2.5	1.6	1
	B*15:01:01G	9	9.3	1
	B*15:03:01G	0	0.3	1
	B*15:07:01G	0	0.3	1
	B*15:14	0	0.3	1
	B*15:34	0	0.3	1
	B*18:01:01G	3	6.5	1
	B*18:01:20	0	0.3	1
	B*18:03	1	0.5	1
	B*27:02:01	0.5	1	1
	B*27:05:02G	3	2.9	1
	B*27:07:01G	0	0.3	1
	B*35:01:01G	7	7.5	1
	B*35:02:01G	0	0.8	1
	B*35:03:01G	1.5	2.9	1
	B*35:08:01	1	0.5	1
	B*37:01:01G	1	1.8	1
	B*38:01:01	5	2.3	1
	B*39:01:01G	1.5	1.6	1
	B*39:05:01	0.5	0	1
	B*39:06:02	1.5	0.8	1
	B*39:09:01	0.5	0	1
	B*40:01:01G	4.5	4.7	1
	B*40:01:18	0	0.3	1
	B*40:02:01G	2.5	1	1
	B*41:01:01	0	0.3	1
	B*41:02:01G	0.5	0.8	1
	B*44:02:01G	7.5	8.6	1
	B*44:03:01G	7	4.4	1
	B*44:03:02G	0.5	0	1
	B*44:05:01	1	0.3	1
	B*44:76	0	0.3	1
	B*45:01:01G	0	0.8	1
	B*48:01:01G	0.5	0	1
	B*49:01:01G	0.5	2.3	1
	B*50:01:01G	1	0.8	1
	B*51:01:01G	7.5	3.6	1
	B*51:07:01	0	0.3	1
	B*51:08:01	1	0.3	1
	B*52:01:01G	1.5	0.8	1
	B*55:01:01G	0.5	1.6	1
	B*56:01:01G	0.5	0.5	1
	B*57:01:01G	2	2.9	1
	B*58:01:01G	0	0.8	1
HLA‐C	C*01:02:01G	2.5	3.4	0.98
	C*02:02:02G	8.5	4.2	0.5
	C*03:03:01G	7	6	0.98
	C*03:04:01G	9.5	10.1	1
	C*04:01:01G	11.5	14	0.9
	C*05:01:01G	6	6.2	1
	C*06:02:01G	7.5	7.5	1
	C*07:01:01G	11.5	16.1	0.9
	C*07:02:01G	11	13.5	0.9
	C*07:04:01G	1.5	2.3	0.98
	C*07:214	0.5	0	0.9
	C*08:02:01G	2.5	1.6	0.96
	C*08:03:01G	0.5	0	0.9
	C*12:02:01G	1.5	0.8	0.9
	C*12:03:01G	7.5	8	1
	C*14:02:01G	0.5	1.3	0.98
	C*14:11	0.5	0	0.9
	C*15:02:01G	3	0.8	0.5
	C*15:05:01G	0.5	0.5	1
	C*16:01:01G	4	2.6	0.9
	C*16:02:01G	2	0.3	0.5
	C*17:01:01G	0.5	1	0.98
HLA‐DP	DPA1*01:03:01G	44	42.4	1
	DPA1*01:04	0.3	0	1
	DPA1*01:06:01	0.5	0	0.96
	DPA1*01:08	0.3	0.4	1
	DPA1*02:01:01; DPA1*02:01:08	2.5	3	1
	DPA1*02:01:02	1.3	1.7	1
	DPA1*02:01:03	0.5	0.9	1
	DPA1*02:01:06	0	0.1	1
	DPA1*02:02:02	0.5	1.2	1
	DPA1*02:02:05	0.3	0.4	1
	DPB1*01:01:01G	2.3	1.7	1
	DPB1*01:01:02; DPB1*162:01	0	0.1	1
	DPB1*02:01:02G	10.3	6.6	0.49
	DPB1*02:01:04	0.3	0	1
	DPB1*03:01:01G	5.5	4	1
	DPB1*04:01:01G	16.8	23.8	0.18
	DPB1*04:02:01G	6.3	6.6	1
	DPB1*05:01:01G	0.8	0.8	1
	DPB1*06:01	0.8	0.8	1
	DPB1*09:01:01	0.3	0.8	1
	DPB1*10:01	0.8	0.7	1
	DPB1*11:01:01G	1	0.7	1
	DPB1*13:01:01G	1.3	0.8	1
	DPB1*14:01:01	0.8	0.5	1
	DPB1*15:01:01G	0.3	0.7	1
	DPB1*16:01:01G	0.3	0.4	1
	DPB1*17:01:01G	1.3	0.3	0.54
	DPB1*19:01:01G	0.8	0.4	1
	DPB1*20:01:01G	0.5	0.1	1
	DPB1*23:01:01G	0	0.1	1
	DPB1*269:01	0.3	0	1
	DPB1*299:01	0	0.1	1
	DPB1*89:01	0	0.1	1
HLA‐DQ	DQA1*01:01:01G	9	8.2	0.87
	DQA1*01:02:01G	7.3	8.9	0.87
	DQA1*01:03:01G	5.8	4.2	0.87
	DQA1*01:10	0	0.1	1
	DQA1*02:01	7.8	5.1	0.87
	DQA1*03:01:01G	5.8	8.3	0.87
	DQA1*04:01:01G	2.5	1.7	0.87
	DQA1*05:01:01G	11.5	13.3	0.87
	DQA1*06:01:01G	0.5	0.3	0.87
	DQB1*02:01:01G	10.5	9.3	0.87
	DQB1*03:01:01G	9.8	10.1	1
	DQB1*03:02:01G	4	4.8	0.87
	DQB1*03:02:02	0	0.1	1
	DQB1*03:03:02G	1.3	2.3	0.87
	DQB1*03:04:01G	0	0.3	0.87
	DQB1*04:02:01G	2.5	1.7	0.87
	DQB1*05:01:01G	7.3	6.2	0.87
	DQB1*05:02:01G	1.5	1	0.87
	DQB1*05:03:01G	1.8	2.1	0.98
	DQB1*05:04:01G	0	0.1	1
	DQB1*06:01:01G	0.5	0.3	0.87
	DQB1*06:02:01G	4	5.8	0.87
	DQB1*06:03:01G	5.5	4.4	0.87
	DQB1*06:04:01G	1	1.3	0.98
	DQB1*06:09:01G	0.5	0.1	0.87
HLA‐DR	DRB1*01:01:01G	3.8	3.4	1
	DRB1*01:02:01G	0.8	0.3	0.88
	DRB1*01:03	0.2	0.1	1
	DRB1*03:01:01G	2.7	4	0.88
	DRB1*04:01:01	1.5	3	0.88
	DRB1*04:02:01	0.2	0.1	1
	DRB1*04:03:01	0.5	0.1	0.88
	DRB1*04:04:01	0.5	0.9	1
	DRB1*04:05:01	0.2	0.3	1
	DRB1*04:07:01G	0.7	0.2	0.88
	DRB1*04:08:01	0.2	0.3	1
	DRB1*04:81N	0	0.1	1
	DRB1*07:01:01G	5	3.1	0.88
	DRB1*07:10N	0	0.3	1
	DRB1*08:01:01G	1.8	1	0.88
	DRB1*08:03:02	0.2	0.1	1
	DRB1*08:04:01	0	0.2	1
	DRB1*09:01:02G	0	0.5	0.88
	DRB1*10:01:01	0	0.4	0.88
	DRB1*11:01:01G	1.8	2.5	1
	DRB1*11:02:01	0	0.1	1
	DRB1*11:03:01	0.5	0.2	1
	DRB1*11:04:01G	1	0.7	1
	DRB1*11:06:01G	0.2	0	1
	DRB1*11:08:01	0	0.1	1
	DRB1*11:169N	0	0.1	1
	DRB1*12:01:01G	0.8	0.7	1
	DRB1*13:01:01G	3.5	2.5	0.94
	DRB1*13:02:01	1	1	1
	DRB1*13:03:01G	0.7	0.6	1
	DRB1*13:05:01	0	0.1	1
	DRB1*13:113N	0	0.1	1
	DRB1*14:01:01G	0.8	1.4	1
	DRB1*14:04:01	0.2	0	1
	DRB1*14:07:02	0.2	0	1
	DRB1*15:01:01G	3	4.4	0.88
	DRB1*15:02:01	0.3	0.2	1
	DRB1*15:13	0.3	0.2	1
	DRB1*16:01:01	0.7	0.5	1
	DRB1*16:02:01	0.2	0.1	1
	DRB3*01:01:02G	19.7	21.2	1
	DRB3*01:15	0	0.2	1
	DRB3*02:01:01G	0	0.1	1
	DRB3*02:02:01G	12.3	10.3	0.88
	DRB3*02:18	0	0.1	1
	DRB3*02:26	0	0.1	1
	DRB3*03:01:01G	1.3	1.5	1
	DRB4*01:01:01G	10.3	9.7	1
	DRB4*03:01N	23	23.7	1

Our study, encompassing a large cohort of VITT patients and controls, demonstrates a lack of significant association between VITT risk and common HLA alleles. These findings contrast with the recent report by Petito et al.,^7^ which included fewer VITT patients and used a different control group. Although both cases and controls were of Caucasian ancestry, subtle regional differences in HLA frequencies among our VITT cohort (recruited throughout Germany) and control (recruited in the north‐east of Germany) cannot be fully excluded. The discrepancy may also reflect differences in cohort size, population structure or statistical power and highlights the need for replication of HLA associations in larger, independent VITT cohorts.

Although we found an overrepresentation of HLA A*31:01:02G, this allele was only present in 12% of VITT patients and therefore cannot be a major risk factor for the immune response against adenovirus resulting in high titre anti‐PF4 antibodies. This indicates that the risk for pathological anti‐PF4 immune response in VITT is not associated with a certain HLA type, as has also been shown for the most frequent pathological anti‐PF4 immune response in heparin‐induced thrombocytopenia.^15^ The lack of an association of VITT with a specific HLA type might indicate that γδ T cells are involved or that T‐cell‐independent B‐cell activation may play a role. Heparin‐induced thrombocytopenia has been linked to marginal zone B cells (16). Marginal zone B cells act as critical ‘first responders’ in the spleen, specifically defending against blood‐borne encapsulated bacteria such as Streptococcus pneumoniae or Neisseria meningitidis. They bridge the gap between innate and adaptive immunity by mounting rapid responses without the typical delays associated with T‐cell help. This may also explain why the anti‐PF4 antibody titres in VITT decline within months (17), despite VITT being a secondary immune response.

As we derived the HLA types from whole genome analysis, we cannot exclude the possibility that interactions between multiple HLA loci in certain HLA haplotype combinations may contribute to VITT susceptibility. However, our data suggest that common HLA alleles do not play a major role in determining the risk for VITT. Further research is needed to fully elucidate the immunological mechanisms underlying VITT and to identify potential biomarkers for risk stratification.

## AUTHOR CONTRIBUTIONS

Linda Schönborn, Thomas Thiele and Andreas Greinacher were responsible for recruiting the VITT patients, interpreted the data and wrote the manuscript. Ana Tzvetkova and Lars Kaderali performed the bioinformatics analysis. Uwe Völker and Sabine Ameling were responsible for the SHIP study and DNA preparation of VITT patients. Sören Franzenburg performed the whole genome sequencing. All authors revised and accepted the final version of the manuscript.

## FUNDING INFORMATION

The study was funded by Deutsche Forschungsgemeinschaft: 374031971‐TRR240, GR 2232/9‐1, SCHO 2052/1‐1, TH 2320/3‐1 and the DFG Research Infrastructure NGS_CC (project 407495230) as part of the Next Generation Sequencing Competence Network (project 423957469). Sequencing was carried out at the Competence Centre for Genomic Analysis (Kiel). Dr. Schönborn was supported by the Else Kröner‐Fresenius Stiftung (‘Wiedereinstiegsförderung für forschende Ärztinnen und Ärzte’), the American Society of Hematology with the ASH Global Research Award and within the Gerhard Domagk Research Program by the Universitätsmedizin Greifswald. Some parts of the study were funded by European Medicines Agency (EMA) service contract No. EMA/2021/17/TDA. The views expressed in this report are the personal views of the authors and may not be understood as representing the position of the European Medicines Agency or one of its Committees or Working Parties.

## CONFLICT OF INTEREST STATEMENT

The authors have no conflicts of interest to disclose.

## Supporting information


Data S1.

## Data Availability

Original data are available upon reasonable request to the corresponding author.
