# Supplementary material for: Lack of association between vaccine‐induced immune thrombocytopenia and thrombosis and HLA loci in a large cohort
Source: Br J Haematol. 2026 Mar 9;209(1):353–7. doi: 10.1111/bjh.70419 (PMC13340469; doi:10.1111/bjh.70419)
Supplement: Supplementary file 1 — Data S1. [file BJH-209-353-s001.docx]

Long title: **Lack of Association Between Vaccine-Induced Immune Thrombocytopenia and Thrombosis and HLA Loci in a Large Cohort**

*Author names*: Linda Schönborn^1*^, Ana Tzvetkova^2*^, Thomas Thiele^1^, Uwe Völker^3^, Sabine Ameling^3^, Sören Franzenburg^4^, Lars Kaderali^4^, Andreas Greinacher^1^

* Linda Schönborn and Ana Tzvetkova contributed equally

*Author affiliations:*^1^ Department of Transfusion Medicine, Universitätsmedizin Greifswald, Greifswald, Germany
^2^ Department of Functional Genomics, Universitätsmedizin Greifswald, Greifswald, Germany
^3^ Institute of Clinical Molecular Biology, Christian-Albrechts-University of Kiel, Kiel Germany
^4^ Institute of Bioinformatics, Universitätsmedizin Greifswald, Greifswald, Germany

Supplementary Material

16. Zheng Y, Yu M, Podd A, Yuan L, Newman DK, Wen R, et al. Critical role for mouse marginal zone B cells in PF4/heparin antibody production. Blood. 2013;121(17):3484-92.

17. Schönborn L, Thiele T, Kaderali L, Greinacher A. Decline in Pathogenic Antibodies over Time in VITT. N Engl J Med. 2021;385(19):1815-6.
